# Social Influences in Sequential Decision Making

**DOI:** 10.1371/journal.pone.0146536

**Published:** 2016-01-19

**Authors:** Markus Schöbel, Jörg Rieskamp, Rafael Huber

**Affiliations:** Department of Psychology, Economic Psychology, University of Basel, Basel, Switzerland; Arizona State University, UNITED STATES

## Abstract

People often make decisions in a social environment. The present work examines social influence on people’s decisions in a sequential decision-making situation. In the first experimental study, we implemented an information cascade paradigm, illustrating that people infer information from decisions of others and use this information to make their own decisions. We followed a cognitive modeling approach to elicit the weight people give to social as compared to private individual information. The proposed social influence model shows that participants overweight their own private information relative to social information, contrary to the normative Bayesian account. In our second study, we embedded the abstract decision problem of Study 1 in a medical decision-making problem. We examined whether in a medical situation people also take others’ authority into account in addition to the information that their decisions convey. The social influence model illustrates that people weight social information differentially according to the authority of other decision makers. The influence of authority was strongest when an authority's decision contrasted with private information. Both studies illustrate how the social environment provides sources of information that people integrate differently for their decisions.

## Introduction

Individuals often ignore their own opinion in favor of the opinions of others. Early experimental results of Asch and Sherif impressively illustrated how the judgments of others influence individuals’ judgments [1–3]. People sometimes follow the behavior of others even when they provide inaccurate information. The present article focuses on a decision-making problem in which several individuals sequentially make decisions and have the potential to influence each other. This situation has been studied by economists who focused on conformity behavior that results from the cognitive integration of socially inferred information improving individual decisions [4,5]. In contrast, social psychologists have additionally emphasized conformity behavior that is motivated by maintaining or building acceptance and belonging. Following a cognitive modeling approach, we sought to examine to what extent individual decisions are affected by different types of social influence. Specifically, we are interested in how socially inferred information and normative expectations of an authority have an impact on individual decisions.

Imagine a physician confronted with the task of diagnosing a type of flu strain in a patient showing several symptoms. The symptoms speak in favor of Influenza A, but symptoms are only probabilistically related to flu strains. Thus the physician knows that her diagnosis will be correct only with a certain probability. Meanwhile she knows that her colleague has diagnosed a case of the relatively harmless Influenza C in the same patient. What should she do: rely on the symptoms that she has observed or follow her colleague’s judgment? If she follows her colleague’s judgment this would be a typical case of conformity behavior, because she is disregarding the evidence the patient’s symptoms provide. Can such a conformity decision be reasonable?

To explain why people conform it is helpful to distinguish two types of social influence: *normative social influence* and *informational social influence* [6]. Normative social influence describes behavior that has been driven by the desire to achieve a valued, coherent self-identity and to convey a particular impression to others [7]. The influence is based on people’s motivation to gain approval and avoid rejection by conforming with others’ expectations. The physician’s decision to conform may be motivated by the desire to avoid looking ridiculous in front of others because she was incapable of diagnosing the harmless Influenza C. In contrast, informational social influence arises from useful and valid information that another’s opinion or behavior provides to improve a decision or judgment [8,9]. If, for instance, the physician’s colleague was very experienced and potentially had additional information for a diagnosis, this informational influence would lead the physician to the correct inference that her colleague’s diagnosis is very likely correct, making her own conforming decision the best she can do.

Dual-motive views of social influence have already been proposed in several domains, appearing in conformity research [6,10], group polarization research [11,12], and persuasion research [13]. Criticism of such views has mainly focused on the problem of how the two types of influence can be separately measured and, consequently, how they interact [14–16]. In many conformity studies individuals’ behavior is examined under two conditions: In the public condition, individuals act under the surveillance of others, whereas in the private condition, responses are given anonymously. If behavior in the public condition differs from behavior in the private condition, this is usually attributed to salient beliefs of the person being socially influenced by the fact that others will positively evaluate his or her conformity behavior. Nevertheless, normative social influence cannot be excluded in the private condition. Social expectations of others can also emerge when their presence is imagined, so they hold across public and private contexts [13]. Moreover, priming studies have suggested that individuals’ tendency to conform can even arise automatically, outside conscious awareness or voluntary control [17,18].

Whereas normative social influences are difficult to control in experimental settings, the utility of informational social influence for improving one’s decisions or judgments is still poorly understood. Most social influence paradigms create conformity by having the influence source give unrepresentative information and then focusing on incorrect answers as dependent measures. Hence, the analysis of informational social influence is tainted by an overemphasis on normative social influence that leads to incorrect conformity decisions [14,16] and pays little attention to the fact that conformity can also improve behavior. For instance, in situations where individuals have to decide under uncertainty others can provide useful accurate information.

In sum, many social psychologists agree that conformity can result from informational and normative social influence. How the two types influence behavior is often difficult to measure, and whether and how they might work together is an even more complicated question. In the present study we examined a sequential decision-making task that allowed us to identify the different types of social influence on individual behavior. More specifically we examined decision making using the “information cascade paradigm” [4,5].

### Information Cascades and Conformity Behavior

Bikhchandani et al. argued that people’s judgments, in principle, are based on *private* and *public* information [5]. For instance, a person’s own examination of a judgment situation provides access to information others have not obtained, which is private information. In addition, the person can consider information that is commonly available to everyone; this is public information. In a situation in which several individuals make the same decision sequentially, the decisions made by others preceding an individual’s own decision provide public information to that individual. An informational cascade occurs when it is optimal for an individual, having observed others’ preceding decisions, to follow the behavior of the preceding person, ignoring his or her own private information. Bikhchandani et al. showed that such decisions are rational when following a Bayesian analysis of the problem [5], which we demonstrate below.

Anderson and Holt examined whether information cascades actually occur [4]. In their experiment, one of two urns was randomly selected by the experimenter. The two urns contained the same number of balls, but the composition of the balls’ color differed for the urns. For instance, both urns could contain three balls, with two white and one black ball for the first urn (Urn A) and two black and one white ball for the second urn (Urn B). The participants knew the compositions of the two urns but did not know which urn was randomly selected by the experimenter. Participants decided sequentially which of the two urns had been selected. Before making a decision, each participant drew one ball from the selected urn and observed its color, which was not revealed to the other participants (i.e., private information) and the drawn ball was afterward put back into the urn. Thereafter, each participant publicly announced his or her decision. Thus, participants had private information, which was the color of the drawn ball from the chosen urn, and public information, which were the decisions of the preceding participants (but not their private signals). To make a correct prediction, participants could use both types of information.

More precisely, according to a Bayesian analysis of the problem, the posterior probability that Urn A was selected could be determined by applying Bayes’s theorem:

$$p(\text{A}|n_{\text{a}},n_{\text{b}})=\frac{p(n_{\text{a}},n_{\text{b}}|\text{A})p(\text{A})}{p(n_{\text{a}},n_{\text{b}}|\text{A})p(\text{A})+p(n_{\text{a}},n_{\text{b}}|\text{B})p(\text{B})}$$ (1)

where *p*(*n*_a_, *n*_b_ |A) is the likelihood of observing the number *n*_a_ and *n*_b_ of “a” and “b” signals given Urn A was selected, where “a” speaks for Urn A and “b” speaks for Urn B. Signals are either obtained from private draws or inferred from public decisions of others. It is easier to determine the log odds of the posterior probability that Urn A was selected relative to the posterior probability that Urn B was selected. When assuming equal a priori probabilities with which the two urns are selected, the log odds are defined as

$$\text{ln}\frac{p(\text{A}|n_{\text{a}},n_{\text{b}})}{p(\text{B}|n_{\text{a}},n_{\text{b}})}=\sum_{i=1}^{n_{\text{a}}}\text{ln}\frac{p(\text{a}|\text{A})}{p(\text{a}|\text{B})}+\sum_{i=1}^{n_{\text{b}}}\text{ln}\frac{p(\text{b}|\text{A})}{p(\text{b}|\text{B})}$$ (2)

(for details see S1 Text). When the log odds ratio is positive, then the posterior probability that Urn A was selected is larger than the posterior probability that Urn B was selected, whereas a negative ratio makes Urn B more likely to have been selected. Under the assumption of equal priors and equal likelihoods of observing “a” or “b” signals, it can be easily seen with Eq 2 that solely the difference in the number of “a” and “b” signals is decisive (regardless of the absolute number of signals). For more details on the Bayesian solution to this problem see also Phillips and Edwards [19], Grether [20], Anderson and Holt [4], or Hung and Plot [21].

The following example illustrates the Bayesian analysis of the sequential decision problem. Suppose there are three people, named John, Jim, and Jack, facing the decision problem. John draws, unobserved by the others, the first ball and publicly decides for Urn A. After John’s decision, Jim draws a ball and also decides for Urn A. Now it is Jack’s turn. He draws a “b-ball,” which indicates the selection of Urn B, but since John and Jim decided for Urn A, Jack infers that John has drawn an “a-ball,” since he decided for Urn A. In addition, Jack infers that Jim also drew an a-ball, because if he had drawn a b-ball he probably would have decided for Urn B, to avoid being misled by a potential mistake of John. Thus, Jack infers that two a-balls (*n*_a_ = 2) and one b-ball (*n*_b_ = 1) have been drawn and can calculate the log odds for Urn A:

$$\text{ln}\frac{p(\text{A}|n_{\text{a}},n_{\text{b}})}{p(\text{B}|n_{\text{a}},n_{\text{b}})}=\text{ln}\sum_{i=1}^{n_{\text{a}}}\text{ln}\frac{\frac{2}{3}}{\frac{1}{3}}+\text{ln}\sum_{i=1}^{n_{\text{b}}}\text{ln}\frac{\frac{1}{3}}{\frac{2}{3}}=0.69,$$

which are positive, so that Urn A should be selected despite the private signal supporting Urn B. Any subsequent decision makers should also follow the decision of the first and second decision makers, so that an information cascade emerges. If a fourth and fifth person drew b-balls it would be rational for them to decide for Urn A. Thus, although after the fifth person three b-balls and only two a-balls have been drawn, making Urn B the most likely selected urn, all individuals would be acting rationally by selecting Urn A according to a Bayesian analysis of the private and public information available to them.

Anderson and Holt observed a high proportion of individuals’ decisions in line with the illustrated Bayesian updating process [4], which has been replicated by a multitude of empirical studies [21–23]. However, compared to people who use the Bayesian solution, participants in cascade experiments seem to overweight their private information relative to the public information [24–26]. A meta-analysis led Weizsäcker to the overall conclusion that people often overweight their private information in comparison to public social information [27].

However, we think that this conclusion needs to be limited to the artificial cascade paradigm examined. We think that people are often strongly influenced by other people’s behavior in many real-life situations and thus overweight *social* relative to private information. Research illustrating the strong impact of social influences on behavior and decision making is widespread; for an overview, see, for instance, Cialdini and Goldstein [28]. Here we illustrate with the sequential decision-making paradigm described above how the impact of social influence can increase depending on the social context in which it is embedded. Moreover, we follow a cognitive modeling approach to identify the importance people give to private as compared to social information.

### Social influence model

To identify the importance people give to different sources of information we suggest a social influence model. For this model we modify Eq 2 by separating one component containing private from another component containing public information:

$$\text{ln}\frac{p(\text{A}|n_{\text{a}},n_{\text{b}})}{p(\text{B}|n_{\text{a}},n_{\text{b}})}=\beta_{\text{bias}}+\beta_{\text{soc}}\sum_{x\in a_{\text{public}}\cup x\in b_{\text{public}}}f(x)+(2-\beta_{\text{soc}})\sum_{x\in a_{\text{private}}\cup x\in b_{\text{private}}}f(x)$$ (3)

where $f(x)=\text{ln}\frac{p(\text{x}|\text{A})}{p(\text{x}|\text{B})}$. The social importance parameter *β*_soc_ (0< *β*_soc_<2) specifies how much weight a person gives to the social as compared to the private information. In the case of *β*_soc_>1 the decision maker overweights social information, and in the case of *β*_soc_<1 the decision maker overweights private information. The prior weight *β*_bias_ represents any initial bias toward one of the two choice options. When *β*_soc_ = 1 and *β*_bias_ = 0 the social influence model is equivalent to the Bayesian solution expressed by Eq 2. Note that the log odds of Eqs 2 or 3 can be easily retransformed into posterior probabilities by

$$p(\text{A}|n_{\text{a}},n_{\text{b}})=\frac{1}{1+{\text{e}}^{-\text{ln}\frac{p(\text{A}|n_{\text{a}},n_{\text{b}})}{p(\text{B}|n_{\text{a}},n_{\text{b}})}}}$$ (4)

The larger the posterior probability is for one option, the larger the probability that a person chooses this option should be. Accordingly we define the choice probability with which a person chooses an option as a function of the option’s posterior probability of being correct:

$$p_{\text{person}}(\text{A})=\frac{1}{1+{\text{e}}^{\theta x(p(\text{B}|n_{\text{a}},n_{\text{b}})-p(\text{A}|n_{a},n_{b}))}},$$ (5)

where *θ*(10 >*θ* > 0) represents a free sensitivity parameter that specifies how sensitive a person’s response is to the different posterior probabilities. A large sensitivity parameter implies that the option with the higher posterior probability will be chosen with a higher probability.

In sum, the social influence model allows us to quantify the importance given to information inferred from others’ decisions (public social information) relative to private information. By specifying the Bayesian solution as a special case of the model, we can test whether people deviate from the normative solution of probability theory.

In the first study, we did not differentiate between different types of social influence. In the second study, we manipulated the hierarchical rank of a previous decision maker to increase that decision maker’s social influence, and the model allowed us to test whether this manipulation affects the social influence. This was achieved by embedding the rather artificial cascade paradigm in a clinical decision-making context. To do this, we drew on the authority principle, which states that people are willing to follow the suggestions of someone that they see as a legitimate authority [29–31]. The principle works within hierarchical relationships, which are asymmetrical in nature and involve the management of dominance “in ways that maximize the interests of the more dominant individual and limit harm to the less dominant individual” [31]. We understand the authority principle as a specific type of normative social influence, since it is based on the deference to authority norm, which is a prevailing norm in most organizations [28]. However, manipulating normative social influence by confronting participants with a decision of a higher ranked person is a relatively weak induction of normative influence when compared to a much more “pressurizing” homogeneous majority opinion.

We examined the impact of social influence in two experiments by testing to what extent individual decisions are affected by social influences according to the following two hypotheses:

1. The *informational influence hypothesis* follows from a Bayesian view of information usage. This hypothesis states that people try to be as accurate in their judgments as they can be, efficiently inferring information from others’ behavior and integrating the socially inferred information with their own private information to derive a decision. This decision can be the opposite of a decision that is reached from private information alone. Decision makers who behave in a manner consistent with the informational influence hypothesis will make decisions in line with the Bayesian model specified above (i.e., Eq 2). The social influence model allowed us to test whether decision makers weight all available information equally to make a decision, regardless of whether it is private or public information.
2. The *authority influence hypothesis* predicts that people’s behavior will also be influenced by the hierarchical status of other decision makers. In line with the authority principle, people will make decisions that conform to higher ranked others’ decisions more often, even if other available public information and their own private information suggest doing otherwise. Behavior that is consistent with the authority influence hypothesis should be better described by the social influence model, which allows decision makers to give greater weight to the information that is inferred from the behavior of the higher ranked other person.

The aim of the following studies was to test these two hypotheses.

## Study 1

The purpose of Study 1 was primarily to test the informational influence hypothesis. The experimental task was constructed in such a way as to minimize normative social influence on people’s decisions, so that conformity behavior would largely express the informational social influence of others. If people’s decisions were consistent with the Bayesian model, as suggested by Bikhchandani et al. [5], this would indicate that individuals’ decisions reflect a process of rational information integration of privately and socially inferred information. In Study 1 we fit the social influence model to participants’ decisions to see if and how people’s behavior deviates from the Bayesian solution.

The experimental task was similar to that used by Anderson and Holt [4]. However, to increase our experimental control, participants were not confronted with real urns from which balls were drawn. Instead they had to make judgments for a series of hypothetical scenarios (see Huck & Oechssler for a similar experimental procedure [32]). This allowed us to systematically vary the information given to each participant. In contrast, in Anderson and Holt’s experiment participants had to announce their decisions to a group, so that normative social influence cannot be ruled out completely. In Study 1 participants were additionally asked to estimate the probability that their predictions were correct, so that we could compare it to the posterior probabilities derived by the Bayesian model (see Eq 2).

### Method

#### Ethics statement

The study was conducted in accordance with the Declaration of Helsinki and the ethical guidelines of the American Psychological Association. Before the start of the experiment, all participants filled out a written informed consent, which informed them about the goals and the completion of the experiment and clearly indicated that they could abandon the experiment at any time without consequences. Prior to the experiment, the investigator collected the signed informed consent forms. No participant abandoned the experiment. All questionnaire data were entered into our database in an anonymized form such that data could not be assigned to individual subjects.

#### Participants

Forty-two students from different departments at the University of Basel participated in the 30-min experiment. Two participants were excluded from further analysis because they said after the experiment that they had not understood the experimental task. Participants received course credit or a book voucher worth 10 Swiss francs. In addition, participants were informed that one of their decisions would be selected randomly, and if that decision was correct they would be rewarded with 2 Swiss francs. If their corresponding confidence rating lay within the range of ±5% of the Bayesian solution they would receive an additional 2 Swiss francs.

#### Procedure

Participants received a questionnaire with a description of the urn decision scenario featuring two urns, each containing three balls, where Urn A had one black and two white balls and Urn B had two black and one white ball. Participants were instructed that one urn was randomly chosen at the beginning of the task by the experimenter and a maximum of four people had the task of sequentially inferring which of the two urns was randomly chosen. They were told that up to four people each sequentially drew one ball from the selected urn, which they replaced in the urn after they privately observed the ball’s color. Thereafter each person announced which urn he or she considered most likely to have been chosen. Thus each person knew the predicted urn of her or his predecessors (but not the color of their drawn balls). It was also explained that each person in the urn scenario had observed his or her predecessors’ decisions. Participants were told they should play the role of the person who made the last decision, in a total of 24 different scenarios.

After the situation description, participants received the 24 scenarios in a randomized order, in which the color of the ball that the last person had drawn and the decisions of the preceding person(s) were provided. The 24 scenarios presented 12 different decision tasks, where all possible combinations of up to four decision makers were specified. Decision sequences where participants were confronted with an unreasonable preceding decision (according to the Bayesian solution) were not included in our scenarios. The 12 decision tasks were presented in two different ways; that is, the decision sequences were mirrored in terms of the color of the balls and the decisions of the preceding people. Thus, each participant decided twice on the same decision task. Participants were asked to predict for each scenario which urn (A or B) was most likely to have been randomly chosen by the experimenter. In addition, they had to judge the probability with which they thought their decision was correct (on a scale of 50–100%). Tables 1 and 2 together summarize the 12 decision tasks with the corresponding posterior probabilities.

**Table 1:** Participants’ decisions and probability judgments for the nine decision scenarios of Study 1 in which, according to a Bayesian solution, the posterior probability of one urn being chosen was above 0.50.

Scenario	Previous decision(s)	Private information favors	Posterior probability	Choices for the most likely urn (%)^a^	Average probability judgment
1	Urn A	Urn A	0.80 for A	91.3	0.66
2	Urn A; Urn A	Urn A	0.89 for A	90.0	0.74
3	Urn A; Urn B	Urn A	0.67 for A	91.1	0.62
4	Urn A; Urn A; Urn A	Urn A	0.89 for A	85.0	0.75
5	Urn A; Urn B; Urn A	Urn A	0.80 for A	85.0	0.69
6	Urn A; Urn A	Urn B	0.67 for A	71.3	0.54
7	Urn A; Urn B	Urn B	0.67 for B	95.0	0.66
8	Urn A; Urn A; Urn A	Urn B	0.67 for A	79.7	0.65
9	Urn A; Urn B; Urn B	Urn B	0.80 for B	93.8	0.74

^a^ Most likely according to the Bayesian analysis

**Table 2:** Participants’ decisions and probability judgments for the three decision scenarios in Study 1 in which a Bayesian analysis led to an indifference situation (i.e., the posterior probability for both urns being 0.50).

Scenario	Previous decision(s)	Private information favors	Posterior probability	Choices for the urn favored by private signal (%)	Average probability judgment
10	Urn A	Urn B	0.50	90.0	0.60
11	Urn A; Urn B; Urn B	Urn A	0.50	65.0	0.60
12	Urn A; Urn B; Urn A	Urn B	0.50	84.8	0.58

### Results

We first analyzed whether participants' decisions were in line with the Bayesian solution. The fifth column of Table 1 shows the proportion of choices in line with the Bayesian solution (see Eq 1). For all tasks in which the posterior probability was in favor of one alternative (Scenarios 1–9), 86.9% of all choices were consistent with the Bayesian prediction. In particular, when the Bayesian prediction was in favor of a participant’s private signal, 90.2% of all choices were consistent with the prediction. To determine whether information cascades occurred, Scenarios 6 and 8 are crucial. Here the Bayesian solution predicted that the private signal should be disregarded in favor of the previous decisions. A high degree of cascade behavior consistently occurred: Of all 160 choices, 120 (75.5%) were consistent with the Bayesian prediction.

In situations with posterior probabilities of *p* = 0.50 (Scenarios 10–12), private and public information canceled each other out. These scenarios allowed us to test whether public social information has a stronger influence than private information. As shown in Table 2, in 79.9% of all choices, participants decided in line with their private signal, thus giving more weight to their own information than to the public information. In sum, the results show that participants used the information provided by others’ decisions in a way that is consistent with a Bayesian analysis of the decision problem, supporting the informational influence hypothesis.

To examine in more detail how much weight participants gave to public information relative to private information, we estimated the importance (*β*_soc_), the bias (*β*_bias_), and the sensitivity (*θ*) parameters of the social influence model on the basis of the observed data. We estimated the model by following a Bayesian approach for each participant [33–35]. This approach provides a posterior probability distribution of each of the model's free parameters. For each parameter, we first specified a prior distribution expressing the initial belief in every possible parameter. For the *β*_bias_ parameter we assumed a prior truncated normal distribution with a mean of zero and a standard deviation of 10, truncated at +1 and -1. For the social importance parameter *β*_soc_ we assumed a prior uniform distribution ranging from 0 to 2 (specified by a beta distribution). Likewise we assumed a uniform prior distribution ranging from 0 to 10 for the sensitivity parameter *θ* (specified by a beta distribution). According to the Bayesian approach, the prior distributions are then updated on the basis of the data and the model's likelihood function (i.e., Eq 5). Technically we relied on JAGS [36] through the rjags interface in R [37]. For the sampler we chose a thinning factor of 100 (to minimize autocorrelation) and an initial burn-in of 10,000 (to produce more representative samples from the posterior). The final Markov chains had a net length of approximately 50,000. Group estimates for the parameters of the model were derived by averaging the posterior distributions of all participants (by averaging the results of the Markov chains). The derived distributions of the means can be used to calculate summary statistics, for example, median and 95% highest density interval (HDI), among others.

**Fig 1:**
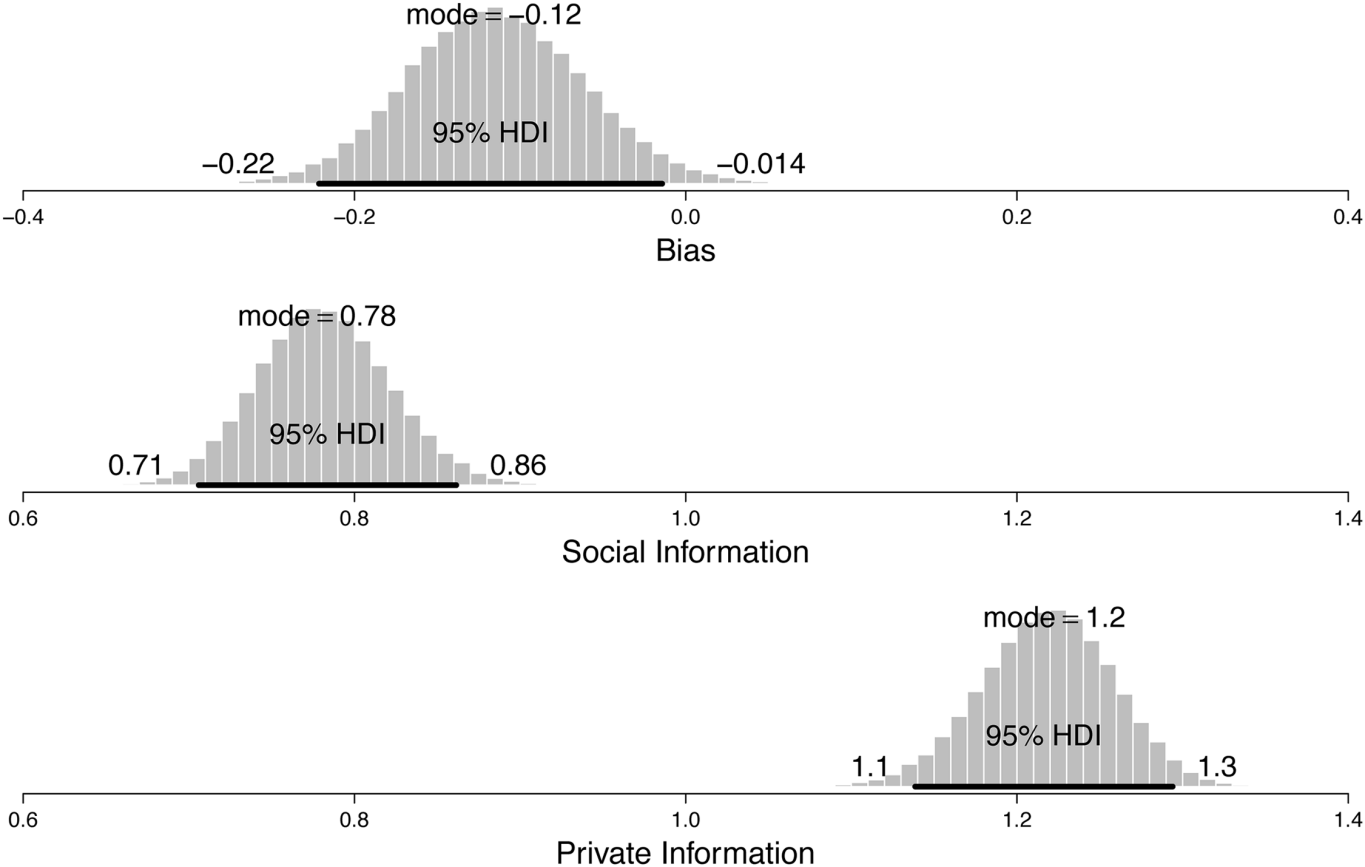
Weighting of public and private information. Marginal posterior distributions for the bias weight, the weight of the public information, and the weight of the private information. The 95% highest density interval (HDI) spans 95% of the posterior distribution.

For the social influence model the median estimated sensitivity parameter was *θ* = 6.08 (95% HDI = 5.57 to 6.58), which implies that participants reacted rather sensitively to the different posterior probabilities. For instance, with a value of 6.08 for the sensitivity parameter, Urn A will be chosen with a probability of 0.89 given a posterior probability of 0.67 for Urn A. For *β*_bias_ the estimated median parameter value was -0.12 (95% HDI = -0.22 to -0.01), which indicates a slight tendency to favor Urn B a priori. Fig 1 illustrates the different weights given to public as compared to private information according to the estimated parameters of the social value model. The median importance parameter given to the public information was *β*_soc_ = 0.78 (95% HDI = 0.71 to 0.86), which shows that participants weighted public information less strongly than private information. When contrasting the weight given to private information (2- *β*_soc_) with the weight given to public information (*β*_soc_) a median positive difference of 0.44 results (95% HDI = 0.28 to 0.59), illustrating overweighting of private information. In sum, the analysis shows that participants overweight private as compared to public information—inconsistent with the Bayesian model that weights all information equally.

In addition to making choices between the two urns the participants had to judge the probability that their choices were correct. The probability judgments, reported in the last columns of Tables 1 and 2, did not match the Bayesian posterior probabilities. Whereas the average probability judgment of 0.59 was higher than the posterior probability of 0.50 in Scenarios 10–12, for scenarios with a posterior probability of 0.67, 0.80, and 0.89 the average probability judgments of 0.61, 0.69, and 0.74, respectively, were lower. These results appear similar to the standard conservatism phenomenon reported in the early literature on probability judgments [38], according to which people tend to give moderate probability judgments. However, our social influence model might give an alternative explanation for these deviations. The social influence model that we propose predicts the probability with which people will select one or the other option (see Eq 5). These predictions follow from the models' predicted *subjective* posterior probabilities that one or the other option is correct. Therefore people's probability judgments can also be compared to these subjective posterior probabilities that the model predicts. Importantly, the model was estimated solely on the basis of participants' choices, ignoring their probability judgments. Therefore predicting participants' probability judgments represents a strong generalization test of the social influence model.

**Fig 2:**
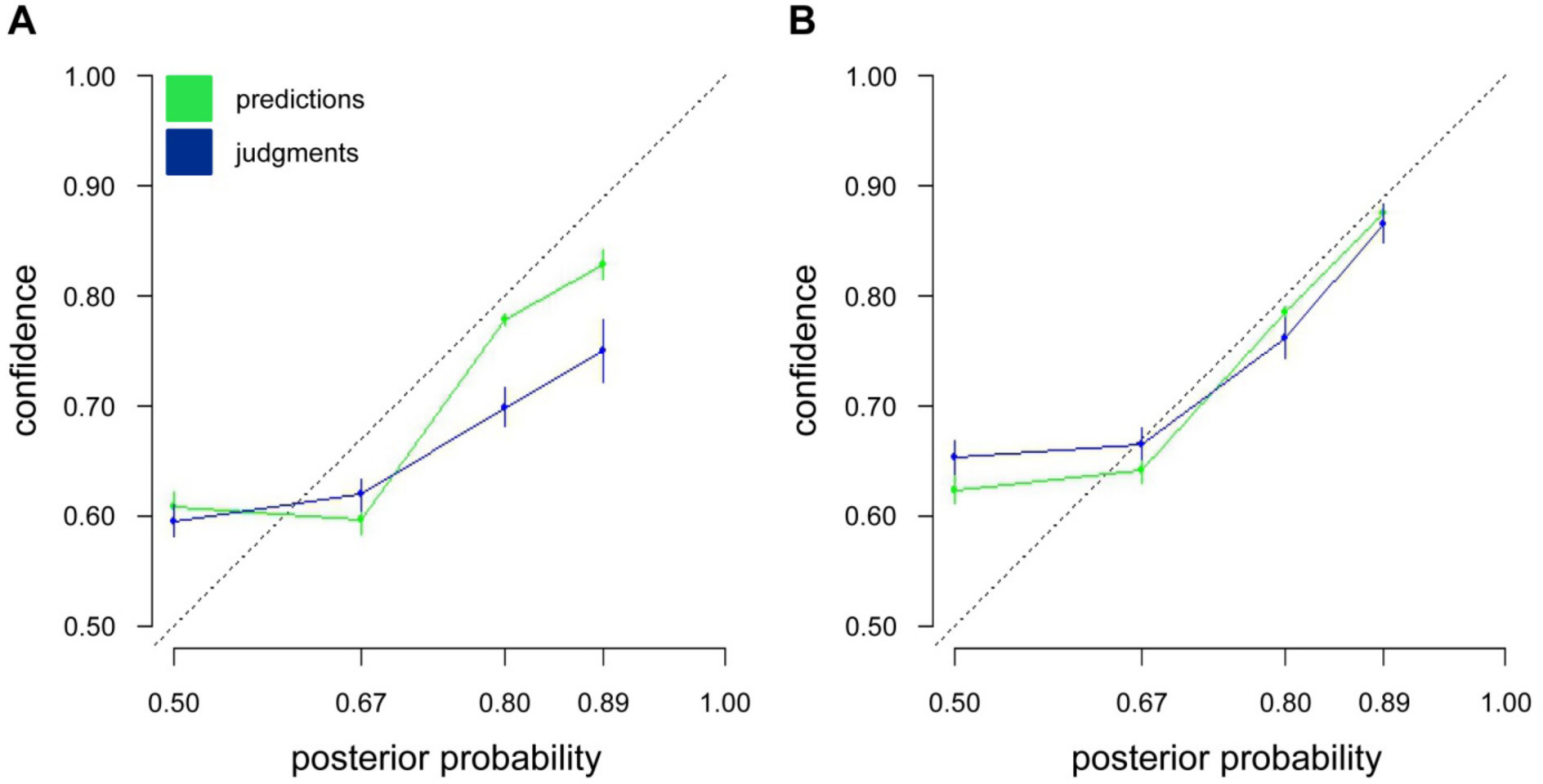
Empirically observed versus predicted probability judgments for Study 1 (A) and Study 2 (B). The general pattern of probability judgments (blue) is accurately captured by the predictions of the model solely derived from participants’ choices (green) for Study 1 (A) and Study 2 (B). Probability judgments on the dashed line are in accordance with the Bayesian solution.

Fig 2A shows that the model predicted the observed probability judgments very accurately. Importantly, the model also predicted overweighting of small probabilities and underweighting of large probabilities. For instance, the model correctly predicted a confidence level of 61% compared to people's observed confidence levels of 59% in situations in which the normative Bayesian account predicts a posterior probability of 50%. Similarly, for situations with a normative posterior probability of 89%, the model predicted a probability judgment of 83% compared to the empirically observed probability judgment of 75%. Thus, the social influence model can predict the observed deviations of people's probability judgments from the normative account. According to the social influence model, these deviations result from overweighting individual as compared to public information. For instance, in the normative indifference situation with posterior probabilities of 50%, overweighting private information leads to increased confidence, whereas in situations with normative high posterior probabilities, overweighting private information leads to more moderate probability levels.

### Discussion of Study 1

Study 1 shows that whether people go against their own private signal depends on whether the posterior probabilities speak for or against their private signal. In situations where private and public information cancelled each other out, participants preferred private information over public information. These results suggest that private information and socially inferred information are cognitively integrated. Furthermore, the results replicate Anderson and Holt’s findings [4], where the participants made real draws from urns. Our hypothetical scenarios have the advantage of maximizing experimental control. For instance, the scenarios minimize potential normative social influences of other people present in a public setting. Therefore, our results illustrate the impact of informational social influence leading to conformity behavior. The results of the social influence model show that participants overweight private as compared to public information, in contrast to the equal weighing of the Bayesian model. Likewise, participants’ probability judgments did not correspond to the Bayesian solution. These deviations from the Bayesian posterior probabilities are explained by the social influence model, according to which people overweight their private information as compared to social information, which on average in the tested situations led to more moderate probability judgments. Importantly, the model predicted these deviations from the Bayesian account without being fitted to the observed probability judgments.

## Study 2

The purpose of Study 2 was to investigate decision making in a real-life situation in which both informational and authority influences can affect people’s decisions. Therefore, in Study 2 the decision problem of Study 1 was embedded in a medical decision-making context. Participants had to take on the role of an assistant physician who had to diagnose, on the basis of particular symptoms, which of two diseases a patient had developed. The task was analogous to that of Study 1: The assistant physicians had information about others’ decisions: here, the previously made diagnoses of other physicians recorded in the patient’s record. The other physicians’ decisions were often not supported by the private information available to the assistant physician. Again, these decisions represent informational social influence to the assistant physician. To examine the social influence of the hierarchical status of the preceding decision makers, the cascade paradigm offers the opportunity to control the strength of authority influence by varying the hierarchical ranking of the preceding decision makers. At the same time, we can control the strength of informational influence by determining the validity of available information that the decision makers in a sequence draw on. In the following, we explain how we manipulate both types of social influence to examine their relative influence.

To manipulate authority influence, the hierarchical ranking of the influence source was varied: The preceding decisions were made either by a colleague (another assistant physician) with the same hierarchical ranking or by a supervisor (the medical director) with a higher hierarchical ranking. This manipulation varied the strength of the authority influence by focusing on the legitimate power of previous decision makers in relation to the assigned hierarchical ranking of the participant’s role. Although our participants did not expect any negative consequences when deciding against the diagnosis of the medical director, we argue that the tendency to conform should emerge as a result of the perceived hierarchical status difference, in line with priming studies on conformity [17,18]. To control the strength of informational social influence, participants were told that the average accuracy of diagnoses on the specific decision problem was the same for the assistant physicians and the medical director. This allowed us to test the informational influence hypothesis and the authority influence hypothesis within the same task.

In Study 2, 40 scenarios were employed in which participants were confronted with the same 12 decision tasks of Study 1. To test our hypotheses, we created all possible variations of the same decision task in terms of varying hierarchical rankings of the previous decision makers. More specifically, 40 scenarios for all possible decision sequences for up to four decision makers were created, in which the medical director and assistant physicians decided at all positions in the decision sequence with corresponding diagnoses. Again, we excluded scenarios with unreasonable preceding decisions (according to a Bayesian analysis), for example, scenarios where two decisions favoring the same diagnosis were followed by an opposing decision. In sum, we created four scenarios with one previous decision maker (one with the assistant physician and one with the medical director as previous decision makers favoring or opposing participants’ private information), 12 scenarios with two previous decision makers, and 24 with three previous decision makers (see Tables 3–6 for the scenarios used in Study 2).

**Table 3:** Participants’ decisions and probability judgments for the 13 decision scenarios of Study 2 in which the posterior probability of one disease according to a Bayesian analysis was 0.67.

Scenario	Previous diagnosis	Private information favors	Posterior probability	Participants choosing the most likely disease (%)^a^	Participants’ average probability judgment	Average proportion of decisions according to private information	Average probability judgment
Baseline scenarios (no previous decision of the MD)							
1	AP: A, AP: S	A	0.67 for A	95.0	0.70		
2	AP: A; AP: S	S	0.67 for S	92.5	0.66	0.59	0.65
3	AP: A; AP: A	S	0.67 for A	75.0	0.60		
4	AP: A; AP: A; AP: A	S	0.67 for A	75.0	0.66		
Scenarios where the decision of the MD favored participants’ private information							
5	MD: A, AP: S	A	0.67 for A	95.0	0.72	0.94	0.69
6	AP: A; MD: S	S	0.67 for S	92.5	0.68		
Scenarios where the decision of the MD spoke against participants’ private information							
7	MD: A; AP: S	S	0.67 for S	87.5	0.61		
8	AP: A, MD: S	A	0.67 for A	82.5	0.64		
9	MD: A; AP: A	S	0.67 for A	82.5	0.67	0.36	0.66
10	AP: A; MD: A	S	0.67 for A	82.5	0.65		
11	MD: A; AP: A; AP: A	S	0.67 for A	87.5	0.70		
12	AP: A; MD: A; AP: A	S	0.67 for A	87.5	0.72		
13	AP: A; AP: A; MD: A	S	0.67 for A	85.0	0.71		

*Note*. AP = Assistant physician; MD = medical director; A = appendicitis; S = sigmoid diverticulitis.

^a^ Most likely according to the Bayesian analysis.

**Table 4:** Participants’ decisions and probability judgments for the 10 decision scenarios of Study 2 in which the posterior probability of one disease according to a Bayesian analysis was 0.80.

Scenario	Previous diagnosis	Private information favors	Posterior probability	Participants choosing the most likely disease (%)^a^	Participants’ average probability judgment	Average proportion of decisions according to private information	Average probability judgment
Baseline scenarios (no previous decision of the MD)							
14	AP: A	A	0.80 for A	97.5	0.80		
15	AP: A, AP: S; AP: A	A	0.80 for A	95.0	0.77	0.97	0.77
16	AP: A; AP: S; AP: S	S	0.80 for S	97.5	0.75		
Scenarios where the decision of the MD favored participants’ private information							
17	MD: A	A	0.80 for A	97.5	0.83		
18	MD: A, AP: S; AP: A	A	0.80 for A	95.0	0.79	0.97	0.79
19	AP: A, AP: S; MD: A	A	0.80 for A	97.5	0.79		
20	AP: A; MD: S, AP: S	S	0.80 for S	95.0	0.76		
21	AP: A; AP: S; MD: S	S	0.80 for S	97.5	0.77		
Scenarios where the decision of the MD spoke against participants’ private information							
22	AP: A, MD: S; AP: A	A	0.80 for A	90.0	0.72	0.86	0.67
23	MD: A; AP: S; AP: S	S	0.80 for S	82.5	0.63		

*Note*. AP = Assistant physician; MD = medical director; A = appendicitis; S = sigmoid diverticulitis.

^a^ Most likely according to the Bayesian analysis.

**Table 5:** Participants’ decisions and probability judgments for the seven decision scenarios of Study 2 in which the posterior probability of one disease according to a Bayesian analysis was 0.89.

Scenario	Previous diagnosis	Private information favors	Posterior probability	Participants choosing the most likely disease (%)^a^	Participants’ average probability judgment	Average proportion of decisions according to private information	Average probability judgment
Baseline scenarios (no previous decision of the MD)							
24	AP: A; AP: A	A	0.89 for A	100.0	0.84	0.98	0.85
25	AP: A; AP: A; AP: A	A	0.89 for A	97.5	0.86		
Scenarios where the decision of the MD favored participants’ private information							
26	MD: A; AP: A	A	0.89 for A	100.0	0.87		
27	AP: A; MD: A	A	0.89 for A	100.0	0.85		
28	MD: A; AP: A; AP: A	A	0.89 for A	100.0	0.89	1	0.88
29	AP: A, MD: A; AP: A	A	0.89 for A	100.0	0.88		
30	AP: A, AP: A; MD: A	A	0.89 for A	100.0	0.88		

*Note*. AP = Assistant physician; MD = medical director; A = appendicitis; S = sigmoid diverticulitis.

^a^ Most likely according to the Bayesian analysis.

**Table 6:** Participants’ decisions and probability judgments for the 10 decision scenarios in Study 2 in which the posterior probability of both diseases according to a Bayesian analysis was 0.50 (i.e., the posterior probabilities predicted an indifference situation between both diseases).

Scenario	Previous diagnosis	Private information favors	Participants’ diagnosis according to their private information (%)	Participants’ average probability judgment	Average proportion of decisions according to private information	Average probability judgment
Baseline Scenarios (no previous decision of the MD)						
31	AP: A	S	70.0	0.62		
32	AP: A, AP: S; AP: S	A	40.0	0.63	0.57	0.63
33	AP: A; AP: S; AP: A	S	60.0	0.66		
Scenarios where the decision of the MD favored participants’ private information						
34	MD: A, AP: S; AP: S	A	75.0	0.68	0.67	0.69
35	AP: A; MD: S, AP: A	S	60.0	0.69		
Scenarios where the decision of the MD spoke against participants’ private information						
36	MD: A	S	37.5	0.62		
37	AP: A, MD: S; AP: S	A	40.0	0.63		
38	AP: A, AP: S; MD: S	A	30.0	0.66	0.39	0.65
39	MD: A; AP: S; AP: A	S	47.5	0.66		
40	AP: A; AP: S; MD: A	S	37.5	0.68		

*Note*. AP = Assistant physician; MD = medical director; A = appendicitis; S = sigmoid diverticulitis.

Next we structured the scenarios according to the corresponding Bayesian predictions, resulting in four groups of scenarios (scenarios with a posterior probability of 0.50, 0.67, 0.80, and 0.89; see Tables 3–6). This study design allowed us to test both social influence hypotheses. According to the informational influence hypothesis, we should obtain no differences in participants’ decision making and probability judgments in (the four groups of) scenarios where the Bayesian solution is the same. However, according to the authority influence hypothesis, participants’ decisions should vary depending on (a) whether the decision of the higher ranked decision maker (the medical director) supports or speaks against participants’ privately held information and (b) whether the medical director is one of the preceding decision makers or not. Because the informational value of the previous decisions was the same regardless of the hierarchical status of the preceding decision makers, changes in participants’ decision making and probability judgments within a scenario group (i.e., a group of scenarios with the same Bayesian solution) could be traced back to the impact of the hierarchical status of previous decision makers. Therefore, we calculated the average proportion of participants’ decisions in favor of their private information for the following three types of scenarios (within each of the four groups of scenarios, i.e., of scenarios with a posterior probability of 0.50, 0.67, 0.80, and 0.89; see Tables 3–6):

1. Scenarios in which only assistant physicians were the preceding decision makers (baseline condition)
2. Scenarios in which the medical director’s decision supported participants’ private information
3. Scenarios in which the medical director’s decision spoke against participants’ private information

In accordance with the authority hypothesis, we predicted that (a) participants would decide more strongly according to their private information (and would be more confident) when the medical director supported it relative to decisions in the baseline condition (i.e., comparing Scenario b to a); and (b) participants would decide less according to their private information (and would be less confident) when the medical director’s decision spoke against it relative to decisions in scenarios of the baseline condition (i.e., comparing Scenario c to a).

### Method

#### Ethics statement

The study was conducted in accordance with the Declaration of Helsinki and the ethical guidelines of the American Psychological Association. Before the start of the experiment, all participants filled out a written informed consent, which informed them about the goals and the completion of the experiment and clearly indicated that they could abandon the experiment at any time without consequences. Prior to the experiment, the investigator collected the signed informed consent forms. No participant abandoned the experiment. All questionnaire data were entered into our database in an anonymized form such that data could not be assigned to individual subjects.

#### Participants

Forty students from different departments at the University of Basel participated in the experiment, which took approximately 1 h. Participants received course credit or a book voucher worth 10 Swiss francs. In addition, participants were informed that one of their diagnoses would be selected randomly, and if that diagnosis was correct according to the Bayesian solution they would be rewarded with 2 Swiss francs. If their corresponding confidence rating lay within the range of ±5% of the Bayesian solution they would receive an additional 2 Swiss francs.

#### Procedure

First, participants received a description of a hypothetical situation in a hospital. They were asked to imagine themselves in the position of an assistant physician who had to make a decision concerning a patient’s disease. Participants were told about two possible diseases, which were a priori equally likely: sigmoid diverticulitis and appendicitis. Both diseases were probabilistically related to two independently occurring symptoms. Participants were informed that the patient suffered from one of the two symptoms; this constituted the participant’s private information. The first symptom, regurgitation, was more often observed when patients suffered from sigmoid diverticulitis; that is, the conditional probability of observing the symptom when the patient suffered from the disease was *p*(regurgitation|sigmoid diverticulitis) = 0.67, whereas the conditional probability of observing the symptom when the patient suffered from appendicitis was *p*(regurgitation|appendicitis) = 0.33. The second symptom, twinges in the lower left part of the abdomen, was more often observed when patients suffered from appendicitis; that is, *p*(lower abdominal twinges |appendicitis) = 0.67, whereas the symptom was less often observed when patients suffered from sigma diverticulitis; that is, *p*(lower abdominal twinges|sigma diverticulitis) = 0.33.

In addition, the scenarios provided public information concerning the previous diagnoses made by other assistant physicians and/or the medical director, which were recorded in the patient’s record. Participants were informed about the average accuracy of the assistant physicians and the medical director when making an independent diagnosis, that is, a diagnosis without knowing other physicians’ diagnoses. Participants were told that an independent diagnosis of the assistant physician and the medical director was correct in two of three cases (*p* = 0.67). Thus, the decisions of all preceding decision makers (independent of their hierarchical rank) had the same chance of being correct.

After the initial situation was described, 40 decision scenarios were given to the participants in a randomized order. The 40 scenarios provided the participants with the symptom of the patient and the previous diagnoses. Tables 3–6 summarize the 40 decision scenarios with the corresponding posterior probabilities. For each scenario participants were asked to predict which disease (appendicitis or sigma diverticulitis) the patient had developed. In addition, they were asked to judge the probability with which they thought their diagnosis would be correct (on a scale of 50–100%).

### Results

The purpose of Study 2 was to examine individuals’ decision making in relation to the predictions of the informational and authority influence hypotheses. We have broken down our analysis into three parts: First, we present the results of testing the informational influence hypothesis. Next, we describe the results of examining the authority influence hypothesis. Finally, we fit the observed decisions with the social influence model, describing the interplay between informational and authority influences.

#### Informational social influence

To examine whether participants behaved according to the Bayesian analysis of the decision problem, we first analyzed their decisions. The fifth column of Tables 3–5 shows the proportion of participants who made choices in line with the posterior probabilities derived from the Bayesian analysis (see Eq 2). For all scenarios in which the posterior probability was in favor of one disease (Scenarios 1–30), 92.0% of all choices were consistent with the Bayesian prediction. In particular, when the Bayesian prediction was in favor of a participant’s private information, 95.1% of all choices were consistent with the prediction. To determine if informational cascades occurred, Scenarios 3, 4 and 9–13 are crucial (Table 3). Here the Bayesian solution predicts that the private signal should be ignored in favor of the previous decisions. Consistently, a high degree of cascade behavior occurred: Of all 280 decisions, 230 (82.1%) were consistent with the Bayesian prediction.

In situations with posterior probabilities of *p* = 0.50 (Scenarios 31–40), private and public information canceled each other out. As in Study 1, these scenarios allow us to test whether public information has a stronger influence than private information. As shown in Table 6, in only 49.7% of all diagnoses did participants decide in line with their private signal. To explain this result it is important to examine the effect of the authority influence, presented in the next section. Overall, the results show that participants used information provided by others’ decisions in a manner consistent with a Bayesian analysis of the problem, supporting the informational influence hypothesis.

#### Authority influence

To examine the authority influence on participants’ decisions, we first analyzed whether participants in general decided against or with the diagnosis of the medical director. We had 1,119 diagnosis decisions in scenarios where the medical director was one of the preceding decision makers. Of these, 838 (74.89%) were in line with the diagnosis of the medical director. However, to evaluate the impact of authority, it is crucial to focus on participants’ diagnoses and probability judgments with regard to (a) the Bayesian prediction of each decision scenario and (b) the comparison of scenarios with and without the medical director as preceding decision maker supporting or opposing participants’ private information. Therefore, we drew on four scenario groups (see Tables 3–6) in which each scenario had the same posterior probability of one disease (Scenarios 1–30) or the posterior probabilities predicted an indifference situation (Scenarios 31–40).

The authority influence hypothesis predicts that (a) participants should decide more strongly according to their private information (and should be more confident) when the medical director’s decision supports their private information relative to decisions in scenarios of the baseline condition where the medical director was not one of the preceding decision makers. Likewise, (b) participants should make fewer decisions according to their private information (and should be less confident) when the medical director’s decision speaks against their private information relative to decisions in scenarios of the baseline condition.

We began with scenarios for which the posterior probability of one disease according to a Bayesian analysis was 0.67 (see Table 3). The average proportion of participants’ decisions favoring the private information was higher in scenarios where the medical director supported participants’ private information compared to the baseline scenarios where the medical director was not one of the previous decision makers (*z* = -5.12, *p* = 0.001 according to a Wilcoxon signed-rank test). Moreover, we found a lower average proportion of decisions according to private information in scenarios where the medical director decided against participants’ private information compared to the decisions in the baseline scenarios (*z* = -4.85, *p* = 0.001). Participants’ average probability judgments with *M* = 0.69 were higher in scenarios where the medical director’s decision supported participants’ private information compared to the baseline condition with *M* = .65, *t*(39) = -2.54, *p* = 0.015. However, the average probability judgments for scenarios where the medical director’s decision spoke against participants’ private information with *M* = 0.66 were not different from the average probability judgments for the baseline scenarios with *M* = .65, (*p* = 0.07).

Next, we present the results of comparing participants’ decisions in scenarios for which the posterior probability of one disease according to a Bayesian analysis was 0.80 (see Table 4). We found no significant difference in the average proportions of decisions according to the private information between scenarios where the medical director’s decision favored the private information and the baseline scenarios (*p* = 0.65). However, participants decided less often according to their private information in scenarios where the decision of the medical director spoke against their private information compared to their decisions in the baseline scenarios (*z* = -2.23, *p* = 0.026), supporting our authority influence hypothesis. No significant differences in the probability judgments were observed between scenarios where the medical director’s decision favored the private information and the baseline scenarios. However, participants’ decisions against the medical director’s decisions showed significantly lower average probability judgments (*M* = 0.67) compared to the average probability judgments in the baseline scenarios (*M* = 0.77, *t*(39) = 4.36, *p* = 0.001).

In scenarios in which the posterior probability of one disease was 0.89 (Table 5), we found no significant differences in participants’ average proportion of decisions in line with their private information between scenarios where the medical director’s decision corresponded to participants’ private signal and the baseline scenarios (*p* = 0.32), whereas their probability judgments significantly differed between the two conditions, *t*(39) = -3.55, *p* = 0.001, in the direction of a higher confidence for decisions which corresponded with the medical director’s decision.

Last, we analyzed decisions and probability judgments in scenarios where the posterior probabilities for both diseases were the same, with 0.50 predicting indifference for the diagnoses (see Table 6). We found no significant differences between scenarios where the decision of the medical director favored participants’ information and the baseline scenarios. Consistent with the authority influence hypothesis, we found a significantly lower average proportion of participants’ decisions in line with their private information in scenarios in which participants’ private information was not also in line with the medical director’s decision compared to the baseline scenarios where the medical director was not one of the preceding decision makers (*z* = -3.42, *p* = 0.001). Participants’ average probability judgments were significantly higher in scenarios where the medical director supported the private information (*M* = 0.69) compared to the average probability judgments in the baseline scenarios (*M* = 0.63, *t*(39) = -5.18, *p* = 0.001). Moreover, the average probability judgments in scenarios where the decisions of the medical director spoke against participants’ private information (*M* = 0.65) were significantly higher compared to the average probability judgments in the baseline scenarios, *t*(39) = -2.54, *p* = 0.015.

In sum, we found strong empirical evidence for our authority influence hypothesis when comparing participants’ decisions in scenarios without the medical director as preceding decision maker (baseline scenarios) with scenarios in which the medical director’s decision contradicted participants’ private information. Here, the average proportion of decisions according to private information indicates a consistent tendency to follow authority influence. The analysis of the impact of authority influence supporting participants’ private information provides evidence that for scenarios with a posterior probability of 0.67, participants more often decided according to their private information (compared to their decisions in the baseline scenarios), whereas this influence was not observed for scenarios with posteriors of 0.80 and 0.89. This could be due to a ceiling effect, because for the scenarios with high posterior probabilities we had already observed high proportions of decisions in line with private information in the baseline scenarios. However, the probability judgments were consistently higher in scenarios with a supporting decision of the medical director compared to the probability judgments in the baseline scenarios, illustrating an authority influence.

#### The social influence model

Finally, we estimated the social influence model on the basis of participants’ decisions. The goal in Study 2 was to distinguish informational from authority influence. Therefore, we decomposed the public information component within Eq 3 into two components instead of only one—one referring to information from higher ranked decision makers and one referring to information from equally ranked decision makers, providing

$$\begin{array}{l} \text{ln}\frac{p(\text{A}|n_{\text{a}},n_{\text{b}})}{p(\text{B}|n_{\text{a}},n_{\text{b}})}=\\ \beta_{\text{bias}}+\beta_{\text{HR}}\sum_{x\in a_{\text{public-HR}}\cup b_{\text{public-HR}}}f(x)+\beta_{\text{ER}}\sum_{x\in a_{\text{public-ER}}\cup b_{\text{public-ER}}}f(x)+(3-\beta_{\text{HR}}-\beta_{\text{ER}})\sum_{x\in a_{\text{private}}\cup b_{\text{private}}}f(x) \end{array}$$ (6)

where *β*_HR_ refers to the importance given to the information derived from the decisions of the higher ranked (HR) medical director and *β*_ER_ refers to the importance given to the information derived from the decisions of the equally ranked (ER) assistant physicians. In the case of *β*_HR_ = 1 and *β*_ER_ = 1 the social influence model specified by Eq 6 is identical to the pure Bayesian model (see Eq 2). To estimate the four free parameters (*β*_bias_, *β*_HR_, *β*_ER_ and *θ—see* Eq 6) of the social influence model for every participant in Study 2 we applied the same Bayesian approach as used in Study 1 (except that we used a precision (SD = 1/ √*precision*) of 0.01 instead of 0.1 for the prior distribution of *β*_bias_).

**Fig 3:**
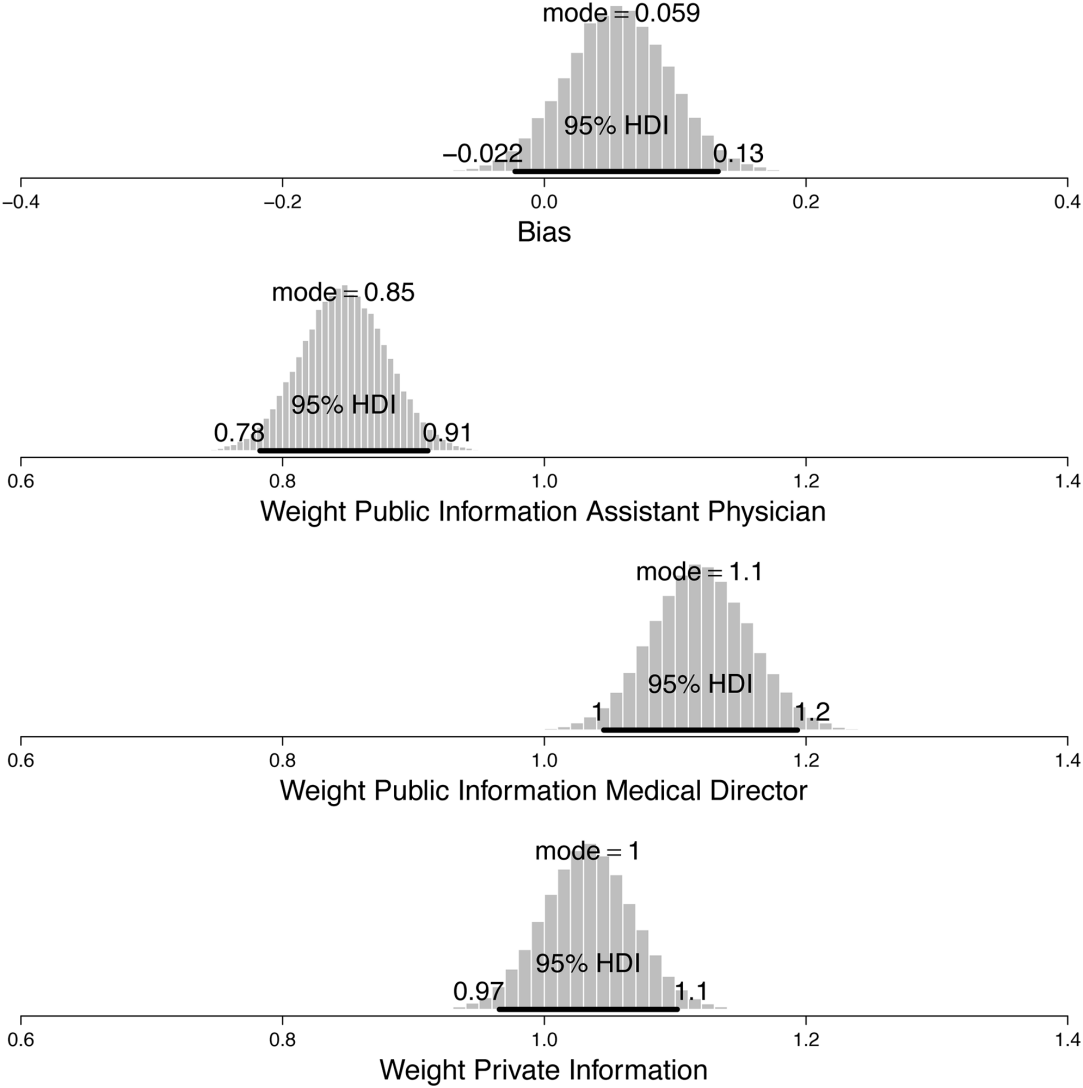
Weighting of different public and private information. Marginal posterior distributions for the bias weight, the weight of the public information derived from the higher ranked physician’s decisions, the weight of public information derived from equally ranked physicians’ decisions, and the weight of the private information. The 95% highest density interval (HDI) spans 95% of the posterior distribution.

The median estimated sensitivity parameter for the social influence model in Study 2 was *θ* = 7.37 (95% HDI = 6.9 to 7.85), thus just a little higher than in Study 1. The median parameter estimate for *β*_bias_ was 0.06 (95% HDI = -0.02 to 0.13), indicating no prior bias toward one of the two decision options. As can be seen in Fig 3, the median importance parameter *β*_HR_ was 1.12 (95% HDI = 1.05 to 1.19), which was higher than the median importance parameter *β*_ER_ = 0.85 (95% HDI = 0.78 to 0.91). The contrast between the two parameters *β*_HR_—*β*_ER_ was positive with a median difference of 0.27 (95% HDI = 0.15 to 0.39). The median weight for the private information of 1.03 (95% CI = 0.97 to 1.10) shows that participants gave more weight to private information than public information derived from the decisions of the equally ranked physicians (*Mdn*_difference_ = 0.19 (95% HDI = 0.08 to 0.30).

However, we found no difference in importance given to private information and information derived from the higher ranked physician (*Mdn*_difference_ = -0.09 (95% HDI = -0.21 to 0.04). Therefore, the results of the social influence model show that people gave greater weight to public information derived from a higher ranked individual than public information derived from equally ranked individuals. Furthermore, in line with the results of Study 1, people overweighted private information when compared to social information derived from equally ranked people.

As in Study 1, we compared the actual to the predicted probability judgments of participants (see Fig 2B). Again, this test of the social influence model was performed purely on the predicted subjective probabilities that were derived from the model, which were estimated on the basis of participants' decisions. Thus, participants' probability judgments were not used at all to fit the model. Again, the social influence model was able to predict people's probability judgments very accurately.

### Discussion of Study 2

The results of Study 2 support the view that individuals are affected by informational and authority influences: The majority of participants made decisions that can be regarded as rational when considering the sequential decision problem from a Bayesian perspective. This held for scenarios in which, according to a Bayesian analysis, the posterior probability of one disease was above .50. Authority influence was observed when the decision of the medical director contradicted participants’ private information (as opposed to the baseline condition), independent of the corresponding posterior probability of the scenarios. The average proportion of decisions according to private information and probability judgments was consistently lower, illustrating the authority influence. With regard to the impact of authority influence supporting participants’ private information, only the analysis of participants’ probability judgments reveals a consistent pattern—that is, higher confidence in their own decision when the previous decision of the medical director was in line with participants’ private information. Finally, the results of our social influence model reveal that people treat public information differently due to its normative quality and independent of its validity. Moreover, the social influence model was also able to predict people's probability judgments quite accurately, importantly without making use of the confidence data to estimate the model's parameters.

## General Discussion

The primary goal of our studies was to examine how individuals’ decisions are influenced by the decisions of others. Therefore, we tried to manipulate informational and authority influences by embedding a social decision task in different contexts. Using the cascade paradigm, we were able to trace back the effects of the two influence types on people’s decisions. Study 1 shows that individuals do integrate socially inferred information to make a decision consistent with a Bayesian analysis. Study 2 shows the impact of authority and informational social influences on individual decision making. Authority influence affects people’s judgments most when the decision of a higher ranked individual speaks against participants’ private information. In these types of situations, people show stronger conformity behavior and lower confidence in their own private information compared to situations in which they are confronted with opposing decisions of individuals whose hierarchical rank is similar to their own. Additionally, we found a consistent authority influence on participants’ probability judgments when previous authority decisions supported participants’ private information.

As a consequence, one can assume that the impact of authority influence should foster the emergence of information cascades. In Study 1 the majority of our participants decided in indifference situations according to their private information (on average 79.9% of all participants, Scenarios 10–12, see Table 2). In Study 2 the majority of our participants decided in indifference situations *against* their private information (on average 61.5% of all participants, Scenarios 36–40, see Table 6) when authority influence was exerted. Given the risk that two decision makers may have unfortunately obtained private information indicating the wrong state of affairs and that subsequent decision makers followed them, the results of Study 2 reveal that only one authority decision will suffice to start a cascade, regardless of subsequent privately obtained information.

The results of Studies 1 and 2 show that people apparently use social information to make decisions in a way that is generally consistent with a Bayesian perspective of the sequential decision problem. However, quantifying social influences with our computational model based on participants’ choices shows that the weight people give to social and private information is context dependent and therefore the weights deviate from the pure Bayesian analysis that weights both kind of information equally. In line with recent studies on cascade behavior [24–27] we found that participants assigned higher weights to private information relative to public information in an urn-and-balls setting (Study 1). In contrast to the procedures used in recent studies on cascade behavior, embedding the decision task in a real-life context reveals that people treat public information derived from higher ranked individuals more seriously than public information derived from equally ranked individuals, whereas they overweight private information as compared to social information derived from lower ranked persons. Therefore, we argue that normative social influence cannot be neglected when analyzing the occurrence of information cascades in real-life settings. Moreover, the model, which was estimated only on the basis of participants' choices, was also able to predict people's probability judgments. For Studies 1 and 2 the model was able to explain why people's probability judgments deviate from the posterior probabilities of the Bayesian account.

The current research sheds new light on the motivational grounds of conformity by clarifying the different roles of informational and authority social influence. The findings of both studies highlight the cognitive aggregation of available public and private information as a decisive factor in the occurrence of conformity. According to the informational influence hypothesis, people evaluate the validity of socially inferred information and integrate it to make a decision. Likewise, one can assume that people are principally influenced by information of others and that authority influence only marginally accounts for conformity behavior. However, in both studies we used a task in which participants’ decisions could be objectively evaluated. Thus the impact of authority influence should have affected people’s decisions less compared to when tasks in which objectively correct solutions are barely, if at all, identifiable (e.g., judging people’s attractiveness [39]). Therefore, the results apply to social influence situations where the intellective properties of a task are salient [12,40,41].

Moreover, our results and conclusions are limited with regard to the operationalization of authority influence. As mentioned above, confronting participants with a decision of a higher ranked person is a relatively weak induction of normative social influence. Thus, following the decision of a higher ranked person may happen for different reasons. For instance, the medical director may be responsible for the assistant physicians’ decisions. In deviating from the director's decision one runs the risk of publically undermining the director's responsibility. Alternatively, following the director's decision may also be driven by the desire to gain the director’s approval, especially when other assistants have decided otherwise. Consequently, future research needs to address these different mechanisms underlying authority influence in more detail.

From an applied perspective, our results reveal that the emergence of informational cascades can be fostered by authority influence. In particular, our results reveal that in situations in which the decisions of higher ranked individuals should have been given the same importance as those of other individuals due to equal decision accuracy, people still assigned more importance to the decisions of the higher ranked individual. Here, the majority of our participants decided against their private information and thereby started a cascade independent of subsequent privately obtained information. From this one may conclude that even when people act rationally according to a Bayesian perspective, a group of decision makers might not make good decisions as a whole. Thus, interventions to support sequential decision-making processes should focus more on changing the design of redundant systems rather than on changing the individual. Here it is important to change the structure of how individuals make decisions. For instance, one can think of systems where individuals first decide without knowing the decisions of their predecessors, after which the single decisions are aggregated in a group context. This has the advantage that all available private information is integrated in the decision of the group.

Improving the reliability of sequential decision-making structures should also include reflections on the incentives that individuals expect. Our studies focused on situations in which people wanted to maximize their individual outcomes; however, social influence situations may differ with respect to their underlying incentive structure. On the one hand, there can be incentives for following the group regardless of being correct. On the other hand, social influence situations can provide incentives to follow the group and make a correct decision. For example, Hung and Plott provided evidence on how information cascades developed when decision makers were positively rewarded when their personal decision was identical to the majority decision [21]. They demonstrated that the attainment of a group goal led to a tendency to place more weight on public information than on private information. Therefore, it seems important to consider the incentives people expect in sequential decision-making structures and whether these goals correspond to their individual goals.

Our studies show that people cognitively integrate both private and public information for making decisions. They attach importance to the inferred information not solely based on its validity but also by taking into account the normative qualities of this information. Therefore, people make smart decisions that aim at being accurate and consistent with their social environment.

## Supporting Information

S1 Text The Bayesian analysis of the sequential decision problem. (DOCX)
