# Supplementary material for: Social Influences in Sequential Decision Making
Source: PLoS One. 2016 Jan 19;11(1):e0146536. doi: 10.1371/journal.pone.0146536 (PMC4718651; doi:10.1371/journal.pone.0146536)
Supplement: S1 Text — (DOCX) [file pone.0146536.s001.docx]

**Supporting Information**

**S1 Text. The Bayesian analysis of the sequential decision problem**

According to a Bayesian analysis the posterior probability of Urn A being selected is determined by applying Bayes’s theorem:

(A1) $p(\left.\text{A}\right|n_{\text{a}},n_{\text{b}})=\frac{p(n_{\text{a}},n_{\text{b}}\left|\text{A})p(\text{A})\right.}{p(n_{\text{a}},n_{\text{b}}\left|\text{A})p(\text{A})\right.+p(n_{\text{a}},n_{\text{b}}\left|\text{B})p(\text{B})\right.}$

where $p(n_{\text{a}},n_{\text{b}}\left|A)\right.$ is the likelihood of obtaining the number *n*_a_ and *n*_b_ of “a” and “b” signals given that Urn A was selected*,* where “a” speaks for Urn A and “b” speaks for Urn B. Analogously, the posterior probability of Urn B being selected given the number *n*_a_ and *n*_b_ of “a” and “b” signals can be determined, so that the ratio of the two posterior probabilities is defined as

(A2) $\frac{p(\text{A}\left|n_{\text{a}},n_{\text{b}})\right.}{p(\text{B}\left|n_{\text{a}},n_{\text{b}})\right.}=\frac{p(n_{\text{a}},n_{\text{b}}\left|\text{A})\right.}{p(n_{\text{a}},n_{\text{b}}\left|\text{B})\right.}\cdot\frac{p(\text{A})}{p(\text{B})}$

Assuming equal a priori probabilities of the two urns being selected and taking the logarithm on both sides provides

(A3) $\ln\frac{p(\text{A}\left|n_{\text{a}},n_{\text{b}})\right.}{p(\text{B}\left|n_{\text{a}},n_{\text{b}})\right.}=\ln\frac{p(n_{\text{a}},n_{\text{b}}\left|\text{A})\right.}{p(n_{\text{a}},n_{\text{b}}\left|\text{B})\right.},$

which can be rewritten as

(A4) **$\ln\frac{p(\text{A}\left|n_{\text{a}},n_{\text{b}})\right.}{p(\text{B}\left|n_{\text{a}},n_{\text{b}})\right.}=\sum_{i=1}^{n_{\text{a}}} \ln\frac{p(\text{a}\left|\text{A})\right.}{p(\text{a}\left|\text{B})\right.}+\sum_{i=1}^{n_{\text{b}}} \ln\frac{p(\text{b}\left|\text{A})\right.}{p(\text{b}\left|\text{B})\right.}.$**
